# 2-Hydr­oxy-10-phenacyl­pyrrolo[2,1-*c*][1,4]benzodiazepine-5,11-dione

**DOI:** 10.1107/S1600536810006902

**Published:** 2010-03-03

**Authors:** S. Ourahou, M. Chammache, H. Zouihri, El Mokhtar Essassi, Seik Weng Ng

**Affiliations:** a Laboratoire de Chimie Organique Hétérocyclique, Pôle de Compétences Pharmacochimie, Université Mohammed V-Agdal, BP 1014 Avenue Ibn Batout, Rabat, Morocco; b CNRST, Division of UATRS Angle Allal Fassi/FAR, BP 8027 Hay Riad, 10000 Rabat, Morocco; c Department of Chemistry, University of Malaya, 50603 Kuala Lumpur, Malaysia

## Abstract

The title compound, C_20_H_18_N_2_O_4_, consists of a benzodiazepinedione system fused to a pyrrole system. The seven-membered ring adopts a boat-shaped conformation (with the methine C atom as the prow); the five-membered ring adopts an enveloped-shaped conformation (with the hydr­oxy-bearing C atom as the flap). In the crystal, the hydr­oxy group is hydrogen bonded to the carbonyl O atom of an adjacent mol­ecule, generating a zigzag chain.

## Related literature

Pyrrolo[2,1-*c*][1,4]benzodiazepines are potent anti­biotics produced by *Streptomyces* species; see: Cargill *et al.* (1974 ▶). For the design of DNA inter-strand cross-linkingand conjugate agents to enhance the sequence selectivity and selectivity for tumor cells, see: Gregson *et al.* (2004 ▶). 
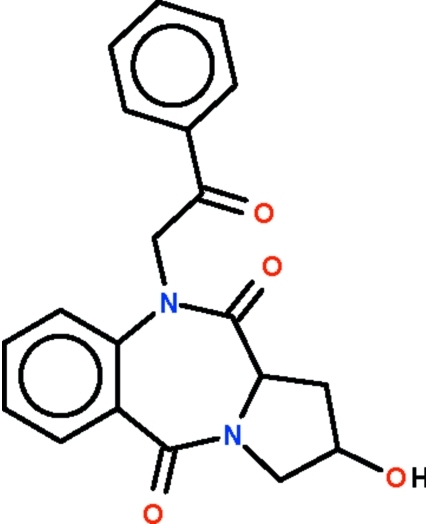


## Experimental

### 

#### Crystal data

C_20_H_18_N_2_O_4_
*M* *_r_* = 350.36
Orthorhombic, 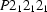

*a* = 8.8337 (2) Å
*b* = 9.9476 (2) Å
*c* = 18.9295 (4) Å
*V* = 1663.41 (6) Å^3^
*Z* = 4
Mo *K*α radiation
μ = 0.10 mm^−1^
*T* = 293 K
0.3 × 0.3 × 0.3 mm

#### Data collection

Bruker APEXII diffractometer
12798 measured reflections
2189 independent reflections
1967 reflections with *I* > 2σ(*I*)
*R*_int_ = 0.030

#### Refinement

*R*[*F* ^2^ > 2σ(*F* ^2^)] = 0.033
*wR*(*F* ^2^) = 0.118
*S* = 1.15
2189 reflections
239 parameters
1 restraint
H atoms treated by a mixture of independent and constrained refinement
Δρ_max_ = 0.31 e Å^−3^
Δρ_min_ = −0.21 e Å^−3^

### 

Data collection: *APEX2* (Bruker, 2005 ▶); cell refinement: *SAINT* (Bruker, 2005 ▶); data reduction: *SAINT*; program(s) used to solve structure: *SHELXS97* (Sheldrick, 2008 ▶); program(s) used to refine structure: *SHELXL97* (Sheldrick, 2008 ▶); molecular graphics: *X-SEED* (Barbour, 2001 ▶); software used to prepare material for publication: *publCIF* (Westrip, 2010 ▶).

## Supplementary Material

Crystal structure: contains datablocks global, I. DOI: 10.1107/S1600536810006902/bt5200sup1.cif

Structure factors: contains datablocks I. DOI: 10.1107/S1600536810006902/bt5200Isup2.hkl

Additional supplementary materials:  crystallographic information; 3D view; checkCIF report

## Figures and Tables

**Table 1:** Hydrogen-bond geometry (Å, °)

*D*—H⋯*A*	*D*—H	H⋯*A*	*D*⋯*A*	*D*—H⋯*A*
O3—H3⋯O1^i^	0.84 (1)	2.02 (2)	2.810 (2)	157 (4)

Symmetry code: (i) 
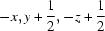
.

**Fig. 1.**
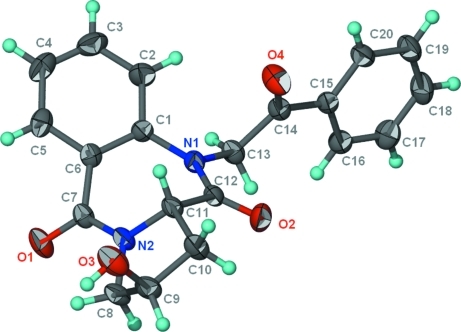
Anisotropic displacement ellipsoid plot (Barbour, 2001) of C20H18N2O4 at the 50% probability level; hydrogen atoms are drawn as arbitrary radius.

## supplementary crystallographic information

### Experimental

2-Hydroxy-pyrrolo[2,1-*c*][1,4]benzodiazepine-5,11-dione (2 g, 8.62 mmol), phenacyl bromide (1.7 g, 8.62 mmol), potassium carbonate (2.4 g, 17.24 mmol) and a catalytic quantity of tetra-*n*-butylammonium bromide was stirred under mild reflux in *N*,*N*-dimethylformamide (60 ml) for 48 h. The insoluble salts were filtered off and the solvent was removed under vacuum. The residue was separated by chromatography on silica gel with an *n*-hexane:ethyl acetate (3:7) solvent system. The compound was obtained as colorless crystals in 70% yield upon evaporation of the solvent.

### Refinement

Carbon-bound H-atoms were placed in calculated positions (C—H 0.93-0.98 Å) and were included in the refinement in the riding model approximation, with *U*(H) set to 1.2–1.5*U*(C). The oxygen-bound H-atom was located in a difference Fourier map, and was refined isotropically with a distance restraint of O–H 0.84±0.01 Å. Due to the absence of anomalous scatterers Friedel pairs were merged and the absolute configuration was arbitrarily set.

### Crystal data

**Table:**

C_20_H_18_N_2_O_4_	*F*(000) = 736
*M**_r_* = 350.36	*D*_x_ = 1.399 Mg m^−^^3^
Orthorhombic, *P*2_1_2_1_2_1_	Mo *K*α radiation, λ = 0.71073 Å
Hall symbol: P 2ac 2ab	Cell parameters from 4805 reflections
*a* = 8.8337 (2) Å	θ = 3.0–29.4°
*b* = 9.9476 (2) Å	µ = 0.10 mm^−^^1^
*c* = 18.9295 (4) Å	*T* = 293 K
*V* = 1663.41 (6) Å^3^	Block, colorless
*Z* = 4	0.3 × 0.3 × 0.3 mm

### Data collection

**Table:**

Bruker APEXII diffractometer	1967 reflections with *I* > 2σ(*I*)
Radiation source: fine-focus sealed tube	*R*_int_ = 0.030
graphite	θ_max_ = 27.5°, θ_min_ = 2.2°
φ and ω scans	*h* = −11→11
12798 measured reflections	*k* = −12→12
2189 independent reflections	*l* = −24→24

### Refinement

**Table:**

Refinement on *F*^2^	Primary atom site location: structure-invariant direct methods
Least-squares matrix: full	Secondary atom site location: difference Fourier map
*R*[*F*^2^ > 2σ(*F*^2^)] = 0.033	Hydrogen site location: inferred from neighbouring sites
*wR*(*F*^2^) = 0.118	H atoms treated by a mixture of independent and constrained refinement
*S* = 1.15	*w* = 1/[σ^2^(*F*_o_^2^) + (0.0831*P*)^2^] where *P* = (*F*_o_^2^ + 2*F*_c_^2^)/3
2189 reflections	(Δ/σ)_max_ < 0.001
239 parameters	Δρ_max_ = 0.31 e Å^−^^3^
1 restraint	Δρ_min_ = −0.21 e Å^−^^3^

### Special details

**Table:**

Geometry. All esds (except the esd in the dihedral angle between two l.s. planes) are estimated using the full covariance matrix. The cell esds are taken into account individually in the estimation of esds in distances, angles and torsion angles; correlations between esds in cell parameters are only used when they are defined by crystal symmetry. An approximate (isotropic) treatment of cell esds is used for estimating esds involving l.s. planes.

### Fractional atomic coordinates and isotropic or equivalent isotropic displacement parameters (Å^2^)

**Table:**

	*x*	*y*	*z*	*U*_iso_*/*U*_eq_	
O1	−0.0779 (2)	0.39357 (16)	0.24376 (9)	0.0502 (4)	
O2	0.47486 (17)	0.28910 (19)	0.16152 (10)	0.0518 (4)	
O3	0.1749 (3)	0.72478 (18)	0.14616 (10)	0.0573 (5)	
H3	0.126 (4)	0.779 (3)	0.1712 (18)	0.096 (14)*	
O4	0.4142 (2)	0.07005 (18)	0.02926 (9)	0.0558 (5)	
N1	0.2550 (2)	0.18311 (17)	0.13852 (9)	0.0343 (4)	
N2	0.1470 (2)	0.44132 (16)	0.19462 (9)	0.0352 (4)	
C1	0.1014 (2)	0.18283 (19)	0.11457 (11)	0.0329 (4)	
C2	0.0577 (3)	0.0859 (3)	0.06534 (14)	0.0500 (6)	
H2	0.1291	0.0261	0.0476	0.060*	
C3	−0.0909 (3)	0.0780 (3)	0.04276 (15)	0.0566 (7)	
H3A	−0.1185	0.0117	0.0106	0.068*	
C4	−0.1975 (3)	0.1657 (3)	0.06668 (14)	0.0520 (6)	
H4	−0.2963	0.1613	0.0498	0.062*	
C5	−0.1575 (3)	0.2611 (2)	0.11618 (13)	0.0412 (5)	
H5	−0.2305	0.3202	0.1332	0.049*	
C6	−0.0085 (2)	0.27020 (19)	0.14120 (10)	0.0325 (4)	
C7	0.0178 (2)	0.37199 (19)	0.19761 (11)	0.0338 (4)	
C8	0.1753 (3)	0.5578 (2)	0.23967 (11)	0.0407 (5)	
H8A	0.2312	0.5325	0.2817	0.049*	
H8B	0.0812	0.6006	0.2536	0.049*	
C9	0.2686 (3)	0.6495 (2)	0.19265 (12)	0.0433 (5)	
H9	0.3342	0.7085	0.2206	0.052*	
C10	0.3610 (3)	0.5518 (2)	0.14819 (13)	0.0450 (6)	
H10A	0.4529	0.5260	0.1727	0.054*	
H10B	0.3880	0.5920	0.1032	0.054*	
C11	0.2580 (2)	0.42975 (19)	0.13715 (11)	0.0332 (4)	
H11	0.2078	0.4344	0.0911	0.040*	
C12	0.3410 (2)	0.2959 (2)	0.14577 (11)	0.0342 (4)	
C13	0.3331 (3)	0.0550 (2)	0.14878 (11)	0.0372 (4)	
H13A	0.2586	−0.0142	0.1585	0.045*	
H13B	0.3987	0.0621	0.1897	0.045*	
C14	0.4276 (2)	0.0125 (2)	0.08502 (11)	0.0350 (4)	
C15	0.5352 (2)	−0.10218 (19)	0.09258 (10)	0.0328 (4)	
C16	0.5804 (3)	−0.1537 (2)	0.15741 (12)	0.0389 (5)	
H16	0.5395	−0.1194	0.1989	0.047*	
C17	0.6868 (3)	−0.2567 (3)	0.16037 (15)	0.0503 (6)	
H17	0.7175	−0.2902	0.2039	0.060*	
C18	0.7472 (3)	−0.3094 (3)	0.09913 (16)	0.0546 (6)	
H18	0.8188	−0.3778	0.1014	0.065*	
C19	0.7009 (3)	−0.2605 (2)	0.03459 (15)	0.0500 (6)	
H19	0.7405	−0.2967	−0.0068	0.060*	
C20	0.5961 (2)	−0.1579 (2)	0.03106 (12)	0.0406 (5)	
H20	0.5656	−0.1255	−0.0128	0.049*	

### Atomic displacement parameters (Å^2^)

**Table:**

	*U*^11^	*U*^22^	*U*^33^	*U*^12^	*U*^13^	*U*^23^
O1	0.0493 (9)	0.0446 (8)	0.0566 (9)	−0.0037 (8)	0.0283 (8)	−0.0046 (8)
O2	0.0303 (7)	0.0521 (10)	0.0730 (11)	0.0000 (8)	−0.0009 (8)	−0.0129 (9)
O3	0.0768 (13)	0.0405 (9)	0.0548 (11)	0.0056 (10)	0.0169 (10)	0.0012 (8)
O4	0.0678 (12)	0.0546 (10)	0.0450 (9)	0.0199 (10)	0.0092 (9)	0.0123 (8)
N1	0.0318 (8)	0.0296 (8)	0.0415 (9)	0.0026 (7)	0.0014 (7)	−0.0074 (7)
N2	0.0379 (9)	0.0292 (8)	0.0385 (8)	−0.0043 (7)	0.0131 (8)	−0.0076 (7)
C1	0.0327 (9)	0.0300 (9)	0.0360 (9)	−0.0053 (8)	0.0024 (8)	−0.0021 (8)
C2	0.0461 (12)	0.0484 (13)	0.0555 (14)	−0.0071 (11)	0.0008 (11)	−0.0178 (11)
C3	0.0553 (14)	0.0611 (15)	0.0535 (14)	−0.0201 (14)	−0.0088 (12)	−0.0107 (12)
C4	0.0378 (11)	0.0618 (15)	0.0563 (14)	−0.0151 (12)	−0.0092 (11)	0.0121 (12)
C5	0.0318 (10)	0.0429 (11)	0.0490 (12)	−0.0046 (9)	0.0025 (10)	0.0128 (10)
C6	0.0321 (9)	0.0295 (8)	0.0360 (9)	−0.0046 (8)	0.0062 (8)	0.0051 (8)
C7	0.0352 (9)	0.0281 (9)	0.0381 (9)	0.0006 (9)	0.0097 (9)	0.0017 (7)
C8	0.0507 (12)	0.0329 (9)	0.0386 (10)	−0.0039 (10)	0.0101 (10)	−0.0110 (8)
C9	0.0497 (12)	0.0326 (10)	0.0474 (11)	−0.0085 (10)	0.0093 (11)	−0.0134 (9)
C10	0.0436 (12)	0.0360 (10)	0.0553 (13)	−0.0132 (10)	0.0165 (11)	−0.0136 (9)
C11	0.0335 (9)	0.0300 (9)	0.0362 (9)	−0.0055 (8)	0.0091 (9)	−0.0063 (8)
C12	0.0301 (9)	0.0355 (10)	0.0369 (10)	−0.0017 (9)	0.0060 (8)	−0.0093 (8)
C13	0.0402 (10)	0.0307 (9)	0.0408 (10)	0.0028 (9)	0.0047 (10)	−0.0018 (8)
C14	0.0365 (10)	0.0295 (9)	0.0390 (10)	−0.0022 (9)	0.0040 (9)	−0.0017 (8)
C15	0.0315 (9)	0.0270 (8)	0.0398 (10)	−0.0044 (8)	0.0028 (8)	−0.0048 (7)
C16	0.0387 (10)	0.0365 (10)	0.0415 (10)	−0.0023 (9)	0.0015 (9)	−0.0023 (8)
C17	0.0469 (13)	0.0456 (12)	0.0586 (14)	0.0048 (12)	−0.0040 (12)	0.0076 (11)
C18	0.0435 (12)	0.0422 (12)	0.0781 (18)	0.0112 (11)	0.0071 (13)	−0.0013 (12)
C19	0.0460 (13)	0.0426 (12)	0.0615 (14)	0.0023 (11)	0.0118 (11)	−0.0147 (11)
C20	0.0419 (11)	0.0400 (11)	0.0401 (10)	−0.0025 (10)	0.0065 (9)	−0.0082 (9)

### Geometric parameters (Å, °)

**Table:**

O1—C7	1.235 (2)	C8—H8B	0.9700
O2—C12	1.221 (3)	C9—C10	1.523 (3)
O3—C9	1.421 (3)	C9—H9	0.9800
O3—H3	0.841 (10)	C10—C11	1.531 (3)
O4—C14	1.207 (3)	C10—H10A	0.9700
N1—C12	1.362 (3)	C10—H10B	0.9700
N1—C1	1.430 (3)	C11—C12	1.529 (3)
N1—C13	1.462 (3)	C11—H11	0.9800
N2—C7	1.334 (3)	C13—C14	1.528 (3)
N2—C8	1.460 (2)	C13—H13A	0.9700
N2—C11	1.469 (2)	C13—H13B	0.9700
C1—C2	1.396 (3)	C14—C15	1.492 (3)
C1—C6	1.397 (3)	C15—C16	1.389 (3)
C2—C3	1.383 (4)	C15—C20	1.397 (3)
C2—H2	0.9300	C16—C17	1.391 (3)
C3—C4	1.361 (4)	C16—H16	0.9300
C3—H3A	0.9300	C17—C18	1.379 (4)
C4—C5	1.380 (4)	C17—H17	0.9300
C4—H4	0.9300	C18—C19	1.377 (4)
C5—C6	1.402 (3)	C18—H18	0.9300
C5—H5	0.9300	C19—C20	1.379 (3)
C6—C7	1.490 (3)	C19—H19	0.9300
C8—C9	1.518 (3)	C20—H20	0.9300
C8—H8A	0.9700		
			
C9—O3—H3	107 (3)	C9—C10—H10A	110.7
C12—N1—C1	124.24 (17)	C11—C10—H10A	110.7
C12—N1—C13	116.19 (16)	C9—C10—H10B	110.7
C1—N1—C13	119.21 (17)	C11—C10—H10B	110.7
C7—N2—C8	122.12 (17)	H10A—C10—H10B	108.8
C7—N2—C11	124.18 (16)	N2—C11—C12	108.02 (17)
C8—N2—C11	112.35 (16)	N2—C11—C10	103.50 (16)
C2—C1—C6	118.6 (2)	C12—C11—C10	113.01 (18)
C2—C1—N1	118.36 (19)	N2—C11—H11	110.7
C6—C1—N1	122.92 (17)	C12—C11—H11	110.7
C3—C2—C1	120.5 (2)	C10—C11—H11	110.7
C3—C2—H2	119.7	O2—C12—N1	121.3 (2)
C1—C2—H2	119.7	O2—C12—C11	122.6 (2)
C4—C3—C2	121.2 (2)	N1—C12—C11	116.05 (16)
C4—C3—H3A	119.4	N1—C13—C14	113.23 (17)
C2—C3—H3A	119.4	N1—C13—H13A	108.9
C3—C4—C5	119.3 (2)	C14—C13—H13A	108.9
C3—C4—H4	120.4	N1—C13—H13B	108.9
C5—C4—H4	120.4	C14—C13—H13B	108.9
C4—C5—C6	121.0 (2)	H13A—C13—H13B	107.7
C4—C5—H5	119.5	O4—C14—C15	120.62 (19)
C6—C5—H5	119.5	O4—C14—C13	120.4 (2)
C1—C6—C5	119.35 (19)	C15—C14—C13	118.97 (17)
C1—C6—C7	124.96 (18)	C16—C15—C20	118.63 (19)
C5—C6—C7	115.64 (19)	C16—C15—C14	123.39 (18)
O1—C7—N2	121.73 (19)	C20—C15—C14	117.96 (18)
O1—C7—C6	121.22 (19)	C15—C16—C17	120.1 (2)
N2—C7—C6	117.01 (17)	C15—C16—H16	119.9
N2—C8—C9	103.16 (16)	C17—C16—H16	119.9
N2—C8—H8A	111.1	C18—C17—C16	120.5 (2)
C9—C8—H8A	111.1	C18—C17—H17	119.8
N2—C8—H8B	111.1	C16—C17—H17	119.8
C9—C8—H8B	111.1	C19—C18—C17	119.8 (2)
H8A—C8—H8B	109.1	C19—C18—H18	120.1
O3—C9—C8	111.3 (2)	C17—C18—H18	120.1
O3—C9—C10	107.82 (19)	C18—C19—C20	120.2 (2)
C8—C9—C10	103.35 (17)	C18—C19—H19	119.9
O3—C9—H9	111.3	C20—C19—H19	119.9
C8—C9—H9	111.3	C19—C20—C15	120.8 (2)
C10—C9—H9	111.3	C19—C20—H20	119.6
C9—C10—C11	105.27 (17)	C15—C20—H20	119.6
			
C12—N1—C1—C2	136.0 (2)	C8—N2—C11—C12	119.19 (19)
C13—N1—C1—C2	−36.8 (3)	C7—N2—C11—C10	166.1 (2)
C12—N1—C1—C6	−47.7 (3)	C8—N2—C11—C10	−0.9 (2)
C13—N1—C1—C6	139.5 (2)	C9—C10—C11—N2	−20.6 (2)
C6—C1—C2—C3	0.9 (4)	C9—C10—C11—C12	−137.16 (19)
N1—C1—C2—C3	177.4 (3)	C1—N1—C12—O2	−174.6 (2)
C1—C2—C3—C4	1.3 (5)	C13—N1—C12—O2	−1.6 (3)
C2—C3—C4—C5	−2.2 (4)	C1—N1—C12—C11	8.7 (3)
C3—C4—C5—C6	1.0 (4)	C13—N1—C12—C11	−178.27 (17)
C2—C1—C6—C5	−2.1 (3)	N2—C11—C12—O2	−112.3 (2)
N1—C1—C6—C5	−178.35 (18)	C10—C11—C12—O2	1.6 (3)
C2—C1—C6—C7	175.3 (2)	N2—C11—C12—N1	64.4 (2)
N1—C1—C6—C7	−0.9 (3)	C10—C11—C12—N1	178.29 (18)
C4—C5—C6—C1	1.1 (3)	C12—N1—C13—C14	−78.0 (2)
C4—C5—C6—C7	−176.5 (2)	C1—N1—C13—C14	95.4 (2)
C8—N2—C7—O1	−7.9 (3)	N1—C13—C14—O4	−11.8 (3)
C11—N2—C7—O1	−173.6 (2)	N1—C13—C14—C15	168.91 (16)
C8—N2—C7—C6	170.02 (18)	O4—C14—C15—C16	165.7 (2)
C11—N2—C7—C6	4.3 (3)	C13—C14—C15—C16	−15.1 (3)
C1—C6—C7—O1	−140.7 (2)	O4—C14—C15—C20	−12.7 (3)
C5—C6—C7—O1	36.8 (3)	C13—C14—C15—C20	166.57 (19)
C1—C6—C7—N2	41.3 (3)	C20—C15—C16—C17	1.4 (3)
C5—C6—C7—N2	−141.1 (2)	C14—C15—C16—C17	−176.9 (2)
C7—N2—C8—C9	−145.4 (2)	C15—C16—C17—C18	−0.7 (4)
C11—N2—C8—C9	21.8 (2)	C16—C17—C18—C19	−0.5 (4)
N2—C8—C9—O3	82.0 (2)	C17—C18—C19—C20	0.8 (4)
N2—C8—C9—C10	−33.5 (2)	C18—C19—C20—C15	0.0 (4)
O3—C9—C10—C11	−84.2 (2)	C16—C15—C20—C19	−1.1 (3)
C8—C9—C10—C11	33.8 (2)	C14—C15—C20—C19	177.3 (2)
C7—N2—C11—C12	−73.9 (2)		

### Hydrogen-bond geometry (Å, °)

**Table:**

*D*—H···*A*	*D*—H	H···*A*	*D*···*A*	*D*—H···*A*
O3—H3···O1^i^	0.84 (1)	2.02 (2)	2.810 (2)	157 (4)

Symmetry codes: (i) −*x*, *y*+1/2, −*z*+1/2.
